# Developed Hybrid Model for Propylene Polymerisation at Optimum Reaction Conditions

**DOI:** 10.3390/polym8020047

**Published:** 2016-02-10

**Authors:** Mohammad Jakir Hossain Khan, Mohd Azlan Hussain, Iqbal Mohammed Mujtaba

**Affiliations:** 1 Department of Chemical Engineering, Faculty of Engineering, University of Malaya, Kuala Lumpur 50603, Malaysia; jakirkhanbd@gmail.com; 2 UM Power Energy Dedicated Advanced Centre (UMPEDAC); Wisma R & D, University of Malaya, Kuala Lumpur 59990, Malaysia; 3 Chemical Engineering Division, School of Engineering, University of Bradford, Bradford BD7 1DP, UK; I.M.Mujtaba@bradford.ac.uk

**Keywords:** polymerisation, developed model, RSM, CFD, optimum production

## Abstract

A statistical model combined with CFD (computational fluid dynamic) method was used to explain the detailed phenomena of the process parameters, and a series of experiments were carried out for propylene polymerisation by varying the feed gas composition, reaction initiation temperature, and system pressure, in a fluidised bed catalytic reactor. The propylene polymerisation rate per pass was considered the response to the analysis. Response surface methodology (RSM), with a full factorial central composite experimental design, was applied to develop the model. In this study, analysis of variance (ANOVA) indicated an acceptable value for the coefficient of determination and a suitable estimation of a second-order regression model. For better justification, results were also described through a three-dimensional (3D) response surface and a related two-dimensional (2D) contour plot. These 3D and 2D response analyses provided significant and easy to understand findings on the effect of all the considered process variables on expected findings. To diagnose the model adequacy, the mathematical relationship between the process variables and the extent of polymer conversion was established through the combination of CFD with statistical tools. All the tests showed that the model is an excellent fit with the experimental validation. The maximum extent of polymer conversion per pass was 5.98% at the set time period and with consistent catalyst and co-catalyst feed rates. The optimum conditions for maximum polymerisation was found at reaction temperature (RT) 75 °C, system pressure (SP) 25 bar, and 75% monomer concentration (MC). The hydrogen percentage was kept fixed at all times. The coefficient of correlation for reaction temperature, system pressure, and monomer concentration ratio, was found to be 0.932. Thus, the experimental results and model predicted values were a reliable fit at optimum process conditions. Detailed and adaptable CFD results were capable of giving a clear idea of the bed dynamics at optimum process conditions.

## 1. Introduction

Polymer-based materials have been a focal point in researches over the last few decades, due to noticeable advancement in improved material properties, compared to other conventional micro- and macro-level materials [1,2,3]. Among polymer-based materials, polypropylene is considered a high-class thermoplastic polymer resin, generated from olefins [4,5]. The extensive applications, from home appliances to all-encompassing industrial usages, have positioned polypropylene as the leading polymer [6,7]. Numerous traditional materials have been replaced by polypropylene, due to its greater physiochemical properties. Several industrial sectors have directly benefited by using polypropylene and its composites [8]. For example, fuel consumption has been reduced remarkably in the automobile sector by replacing metals with polypropylene, as it is lighter. Other physiochemical properties such as cutting-edged structural stability, superior dielectric vitality, and better corrosion resistance competency, have impressed consumers, and the choice of polypropylene is the best alternative to conventional materials [9,10]. Although it has wide acceptability in the global materials market, polypropylene and polypropylene based materials comprise just 20% of the polyolefin market share. Therefore, from a scientific and economic perspective, it is relevant to conduct research on optimising propylene polymerisation, to increase its application and expand its market share [11].

Multidisciplinary efforts have been made to develop the polymerisation process and its procedures, to better understand the complicated flow behaviours and process parameters which are necessary for improving the reactor performance [12,13]. As an example, the fluidisation technique has been applied commercially and is a well-recognised technology. Excellent mass and heat transfer rates, uniform particle mixing, and an ability to achieve diverse chemical reactions, are some of the special features of fluidised bed reactors [14,15,16]. Gas phase polymerisation has been acclaimed as a more sustainable and user-friendly technology by several researchers. A number of factors such as fluidised bed components, system temperature, and gas-solid alignment, can influence the polymer fluidisation performance. Ironically, all these impelling factors make reaction regime analysis difficult. However, the quality control of different grades of polypropylene is highly correlated with these factors. The exothermic nature and sensitivity to system pressure of the propylene polymerisation reaction, can also be broadly influenced by the overall operating conditions [17]. The development of a valid model to clarify the functional relationship among the process variables is vital, to design a robust reaction system to carry out the reaction safely, and to produce uniform and consistent product quality. The model would also support better decision making in many industrial applications [18,19,20,21].

Statistical modelling with response surface methodology (RSM) has been employed in lab-scale to industrial-scale research, to ascertain the optimum operating conditions of a process by several research groups [22,23]. RSM is typically suitable to solve complexities where the explanation of the process dynamics is indistinct, and it is complicated to justify it by a first-principles mathematical model. Under RSM, the standard factorial and Central Composite Design (CCD) are generally proposed to scrutinise the interactions of process factors, based on polynomial models [22,24]. Alternatively, purely mathematical models have also been described, by assuming the hydrodynamics of the fluidised bed reactor in propylene polymerisation [25,26]. However, it has also been reported that developing a mathematical model for a pilot-scale polyolefin production plant is difficult, as the rate of polyolefin production is very sensitive to the essential process parameters of temperature, pressure, feed concentration, and the geometry of the reaction unit [27].

Correspondingly, the literature does not provide any evidence that any optimisation study has been carried out so far, by considering the integrated process parameters with the CFD method on propylene polymerisation. Although conducting pilot-scale research is very important for any industrial decision making procedure, it is rarely reported. The purpose of this study is to examine the multidimensional approaches (from the statistical and CFD point of views) among specific operating parameters for propylene polymerisation in a real reaction pilot-scale environment, and to identify the optimum process parameters by the combination of a predictive CFD coupled RSM model and experimental validation. The operating parameters that have been chosen are reaction temperature (RT), monomer concentration (MC), and system pressure (SP).

An integrated method for identification of optimum process parameters and dynamic transformations of the bed for propylene polymerisation is described in this paper. The experiments were conducted in a pilot-scale plant which is a prototype of an industrial-scale plant, and is currently in the full-range production facilities under the Malaysian National Petroleum Authority (PETRONAS). The sampling and measurement facilities confirmed the uniqueness of our engineered pilot plant, as this system was integrated with a real time data acquisition system and cutting-edge online sampling capacities by a Refinery Gas Analyser (RGA). On a global scale, this type of pilot plant is very exceptional, although it is demanded in industrial production facilities. As there are no indications to the contrary, we consider this to be unique research on the optimisation of propylene polymerisation by employing RSM and investigational validation, in a novel engineered pilot-scale plant.

One of the main concepts of the hypothesis is to apply the well-recognised central composite design [16,28] to propose easy to understand and industrially applicable optimum process parameters, together with their detailed interaction along with fluidised bed dynamic behaviours. The robustness of the experimental design is also discussed in terms of the composite design, and emphasis on constructing an adequate precision ratio, the analysis of variance (ANOVA), and the significance of second-order models, determined by the *F*-value, normal percentage probability, and an interaction graph. The quadratic model provides better evaluation capability for the response surface, and is given in general and actual equations. The face-centred option was chosen to attain the least possible number of experimental runs and the highest possible 3D value.

## 2. Experimental Study

### 2.1. Description of Experimental Setup

**Figure 1:**
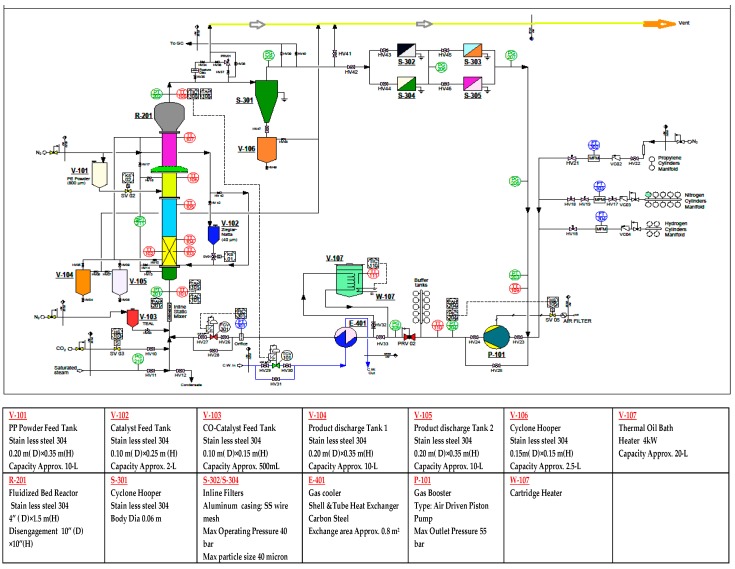
Detailed process diagram of fluidisation of the polypropylene production system.

**Figure 2:**
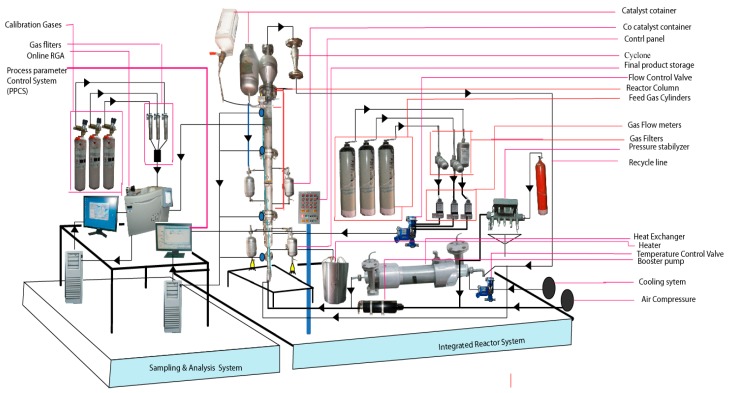
Sampling and analysis system integrated with a pilot scale fluidised bed catalytic reactor.

A pilot-scale fluidised bed catalytic reactor was built to conduct the gas phase polypropylene production, comprising of a fluidised bed and a disengagement section. The detailed schematic diagram and a 3D illustration of the production process are shown in Figure 1 and Figure 2 respectively. The height of the fluidised bed was 150 cm and the diameter was 10 cm. The volume of the disengagement region was fixed at 625 cm^2^. A specially-fabricated catalyst container was installed at a point 9 cm higher than the metallic distributor mesh. The final product haul out points were set at three different heights above the distributor plate. To maintain proper mechanical stability inside the reactor system, the granulated polymer powder was always retained.

As temperature control is a very sensitive issue for fluidised catalytic polymerisation reactions, the system was kept within a 70–80 °C range. To achieve the reaction initiation temperature, a heater was used to heat up the inlet gas mixture. To obtain a detailed temperature profile in the system, six temperature sensors were installed vertically at different points of the pilot plant, starting at 16 cm above the metallic mesh. The unused gases were passed through a heat exchanger to be cooled down, as the mixture had a higher temperature than required. The cyclone was integrated with four filters, equipped to eliminate fines entrained from the reactor. For the purpose of keeping the system pressure always stable, an air plunge compressor was used. A control valve was attached to the reactor system, to regulate the inlet flow and flow circulation inside the reactor. A nitrogen gas cylinder, used as a buffer container, was installed to balance pressure fluctuations. Several gas cylinders of propylene, nitrogen, and hydrogen were used for feedstock loading. The co-catalyst was dosed after confirming and fixing the gas composition. The objective of injecting the co-catalyst was to keep the moisture level below 2 ppm and activate the catalyst, which is a prerequisite for manufacturing commercial grade polypropylene. The mass flow for the co-catalyst was adjusted by the control valve, which revolves at a regular, very fast speed, and injects the co-catalyst into the reactor. In the pilot plant, unreacted gases were recycled through the cyclone and four filters described earlier. The Ziegler-Natta catalyst container was always kept above atmospheric pressure with nitrogen, to avoid contamination. Three different gas purifiers were added to the source line of propylene, hydrogen, and nitrogen, to remove traces of O_2_, H_2_O, CO. Three flow meters were used to measure the flow feed gases. The system was fabricated to withstand a maximum pressure of 30 bar. A relief valve, pre-set at 30 bar to avoid over pressure inside the system, was placed at the top of the reactor.

Propylene, nitrogen, and hydrogen, used as feed gases in the fluidised bed reactor, all work as heat transfer agents. Nitrogen is used as the reactant carrier gas, and hydrogen as the polymer chain disassembly agent. These gases were passed through the distributor flanking the bottom of the reactor. The disengaging region of the rector system is where unreacted gases and solid particles are separated from each other. Fresh feed gases are introduced with the solid-free gases, and recycled back into the system through the metallic mesh. The polypropylene produced is continuously withdrawn from the product discharge line, located at the bottom of the reactor. The propylene polymerisation can fluctuate 2%–3% per cycle, while the complete reaction cycle can produce nearly 98% polymerisation, if the gas–solid fluidisation techniques have been adopted [17].

### 2.2. Measurement and Analysis System

To test the gas composition, the online refinery gas analyser was connected to the sampling line of the reactor system. A set of comprehensive computing equipment and hi-tech data logging tools, were deployed at the pilot plant. The real time data on components of H_2_, N_2_, and propylene, were examined through updated RGA (PerkinElmer Clarus^®^ 580 HYBRID series). Engineering gas chromatography software upgraded by PerkinElmer, USA and University of Malaya, Malaysia, which is capable of analysing a wide-range of hydrocarbons and light gases, investigated the gas composition. The real time data, delivered by this integrated measuring system, were collected. At intervals of 8.5 min, the data acquisition system delivered three types of data, channelled through double Thermal Conductivity Detectors (TCD) and a single Flame Ionisation Detector (FID). The TCD channel displayed data mainly on the carrier gas (nitrogen) and hydrogen. The FID channel provided data for a wide range of hydrocarbons. However, in this study we will only consider the data for propylene.

### 2.3. Model Development for Optimisation

The response surface methodology is an assemblage of both statistical and mathematical approaches that comprise the experimental blueprint, for expressing the scope of the input variables, and observed mathematical model, in order to examine a suitable estimating relationship amid the achieved responses [23,29]. This methodology can also anticipate the optimisation structures, for accomplishing the optimum outputs for the process variables that generate the predicted response [18]. If each independent input parameter (*x*_1_, *x*_2_, *x_k_*) is determinate, governable, and random in the experiment environments, with slight minimum error, then linear yield (response) *Y*_R_ can be expressed as:

(1) $$Y_{R}=f(x_{1},x_{2},...,x_{k})+\varepsilon$$

Additionally, in the RSM the relationships can be given by the polynomial equation expressed as:

(2) $$Y_{R}=\beta_{0}+\sum_{i=1}^{k}\beta_{i}\chi_{i}+\sum_{i=1}^{k}\beta_{ii}\chi_{i}^{2}+\sum_{i=1}^{k-1}\sum_{j=i+1}^{k}\beta_{ij}\chi_{i}\chi_{j}+\int P_{\text{rt}}+\varepsilon$$

where β_0_, β*_i_*, β*_ij_* represent the regression coefficients which might be determined by mathematical model. The value of *P*_rt_ is shown in a later section.

CCD was employed to study the interaction of the process parameters and to predict the optimum polymerisation conditions. After completion of the data acquisition from the experimental study, the next step was to explain an empirical model for the response surface. The level of fit of the polynomial model can be explained by the coefficients of determination *R*^2^ and *R*^2^_adj_, determined by Equations (3) and (4) respectively.

(3) $$R^{2}=1-{\frac{SSQ_{\text{residual}}}{SSQ_{\text{model}}+SSQ}}_{\text{residual}}$$

(4) Radj2=1−SSQresidualDgFresidual(SSQmodel+SSQresidual)/(DgFmodel+DgFresidual)

In Equations (3) and (4), SSQ is the sum of squares, and *DgF* the degrees of freedom from the ANOVA table. The three-factor experiments were conducted at the design centre to evaluate the pure error and were carried in randomised order, as required in many design procedures. Reaction temperature (*A*), system pressure (*B*), and monomer concentration (*C*), were selected as the input process variables. Reaction temperature refers to the temperature maintained at the reaction start-up point, while system pressure refers to the prerequisite pressure inside the system. For fluidised bed polymerisation, a minimum pressure of 20 bar is mandatory for a reaction, although pressure can be raised to 30 bar. The coded value with lowest (−1) and highest (+1) icons and physical properties of the polymerisation process are given in Table 1.

**Table 1:** Range of the independent process variables employed in the experimental design and physical properties of the reaction system.

Code of the factor	Factor name	Units	Type	Low coded	High coded	Low actual	High actual
*A*	Reaction temperature (RT)	°C	Numeric	−1.000	1.000	70.00	80.00
*B*	System pressure (SP)	bar	Numeric	−1.000	1.000	20.00	30.00
*C*	Monomer concentration (MC)	%	Numeric	−1.000	1.000	70.00	80.00
**Physical properties**					**Value**		
Bubble diameter (m)					550 × 10^−4^		
Gas velocity (m/s)					0.50		
Gas density (kg/m^3^)					23.45		
Gas viscosity (Pa s)					1.14 × 10^−4^		
Polymer density (kg/m^3^)					1000		
Void fraction of the bed at minimum fluidisation					0.45		

#### 2.3.1. CFD Modelling of Gas-Solid Phenomenon in FBCR

A two-phase gas–solid model was analysed to explain the fluidised bed dynamic behaviour at optimum process conditions. The commercial software package, ANSYS 16.1 (latest version), was used as it provides integrated and parallel computational facilities for complex multi-phase flows and process parameter optimisations under the options of FLUENT and Design Exploration, respectively. In the present work, in order to simulate a multiphase flow, the Eulerian-Eulerian approach was applied. A built-in model, known as the PBM (population balance model), and a moment method were used to measure the polymer production percentage.

The method includes mathematical evaluation of the emulsion and bubble phases, classifying them as intrusive sequences, whose dynamics is responsible for the value of the production proportion. In the cases when the method of moment and population balance are used, the polymer’s physiochemical properties, including monomer conversion, active site information, and polymer production rate, can be conjectured.

Below is the population balance characteristic of living chains dwelling on active sites, whose dimensions are *r* = 1:

(5) $$\begin{array}{l} \frac{dN(1,\text{s})}{dt}=k_{i}(\text{s})N(0,\text{s})[\text{M}]+Y(0,\text{s})[k_{\text{fm}}(\text{s})[\text{M}]\\ -N(1,\text{s})\times \left[k_{\text{p}}(\text{s})[\text{M}]+k_{\text{fm}}\left(\text{s}\right)[\text{M}]\right]+k_{\text{fst}}(\text{s})+k_{\text{da}}(\text{s})+k_{\text{dl}}(\text{s})[l_{\text{m}}]+\frac{Y_{ppc}}{\sum \zeta_{\text{eb}}} \end{array}$$

Living chains, whose length is more than 1, have a population balance of:

(6) $$\frac{dN(r,\text{s})}{dt}=k_{\text{p}}(\text{s})[\text{M}]N(r-1,\text{s})-N(\text{r},\text{s})\times k_{\text{p}}(\text{s})[M]+k_{\text{fst}}(\text{s})+k_{\text{da}}(\text{s})+k_{\text{dl}}(s)[l_{m}]+\frac{Y_{ppc}}{\sum \zeta_{\text{eb}}}$$

Dead chains are characterised by lengths smaller than 2 and their population balance is:

(7) $$\frac{dQ(r,s)}{dt}=N(r,s)\{[M]k_{fm}(s)+k_{\text{fst}}(s)+k_{\text{da}}(s)+k_{\text{dl}}(s)[l_{m}]+\frac{Y_{ppc}}{\sum \zeta_{\text{eb}}}Q(r,s)$$

By merging Equations (5)–(7) and summing upon all r values, the subsequent mass balance on *Y*(0,*s*) can be obtained:

(8) $$\frac{dY(0,s)}{dt}=[M]\{k_{i}(s)N(0,s)\}-Y(0,s)\{k_{\text{fst}}(s)+k_{\text{da}}(s)+k_{\text{dl}}(s)[l_{m}]+\frac{Y_{ppc}}{\sum \zeta_{\text{eb}}}$$

The equation used in the RSM model has considered the population balance as constant for response calculation purposes. The model has also adopted the notions of multisite polymerisation kinetics and rigorous multi-monomer.

#### 2.3.2. Phase Sequestration

This function was calculated as the volume quadratic mean of the volume fraction (solid–gas) over the bed apportioned by the preliminary static bed height. Greater values of this function measurement specified greater volume fraction oscillations throughout the averaging procedure, and consequently the substantial solids volume fraction showed heterogeneities in the two phases. The phase sequestration measurement is also an indicator of the quality of the gas–solid contact attained in the reactor. A high degree of phase sequestration implies improved contact between the solid and the gas and thereby, an enhancement in the performance of the reactor [29,30] .The necessary correlations involved in the fluidisation of both the phase models are given in Table 2.

It was assumed that propylene consumption took place immediately after the catalyst dosing, where hydrogen depletion transpired due to the engagement of hydrogen in the polymer chain expurgation.

(9) $$\int P_{rt}=\sum_{i=1}^{2}P_{mw}R_{i}$$

*R_i_* is the instantaneous rate of polymerisation.

The mass, momentum and energy interactions between both phases were also taken into account. The energy equation was considered in this case since the flow was in exothermal conditions. Here, the noticeable forces on the particles were the drag and gravity, while the virtual mass and lift effects were neglected due to the higher density ratio of the solid to the gas phase. The standard k–ε turbulence model was used to model the solid phase. It should be highlighted that the granular temperature was solved for each phase. The solid shear viscosity consisted of collisional, kinetic and frictional effects. Schaeffer's expression [28] was used to model the frictional viscosity in the dense cases. The solid pressure consisted of two terms. The first term represented the kinetic term and the second term, which accounted for the particle collisions, was calculated using the Maxwellian distribution. The radial distribution function modified the probability of the particle collisions as the phase became denser [31]. A two-dimensional physical model of the reactor system must be available in order to study the pilot FBR plant. Although it has been pointed out that there are differences between 2D- and 3D-simulated void fractions, the 2D model is still recommended to reduce the cost of calculation while maintaining accuracy [32,33]. In addition, the 2D simulation has always been applied because of much cheaper numerical costs and less computational time [28,34,35]. The next sections describe the main governing equations behind the developed model.

**Table 2:** Dynamic correlations and formulas applied for the CFD model for the bubble and emulsion phase.

Parameter	Formula	Reference
Bubble velocity	$v_{b}=v_{o}-v_{e}+v_{br}$	[36]
Bubble rise velocity	$v_{br}=0.7119{\left(gd_{b}\right)}^{1/2}$	[37]
Emulsion velocity	$v_{e}=\frac{v_{0}-\partial v_{b}}{1-\partial }$	[38]
Bubble diameter	$d_{b}=d_{br}{\left[1+27(v_{0}-v_{e})\right]}^{1/3}(1+6.84H)$ $d_{br}=0.0085$ (Geldard B category)	[39]
Bubble phase fraction	$\partial =0.534(1-e^{-\frac{v_{0}-v_{mf}}{0.413}})$	[40]
Emulsion phase porosity	$\chi_{e}=\chi_{mf}+0.2-0.059e^{-\frac{v_{0}-v_{mf}}{0.429}}$	[40]
Bubble phase porosity	$\chi_{b}=1-0.146e^{-\frac{v_{0}-v_{mf}}{0.439}}$	[40]
Volume of polymer phase in the emulsion phase	$\zeta_{pe}=AH\left(1-\chi_{e}\right)(1-\partial )$	[17]
Volume of polymer phase in the bubble phase	$\zeta_{pb}=AH\left(1-\chi_{b}\right)\partial$	[31]
Volume of the emulsion phase	$\zeta_{e}=AH\left(1-\chi_{b}\right)$	[41]
Volume of the bubble phase	$\zeta_{b}=A\partial H$	[37]
Minimum fluidisation velocity	$\beta e_{mf}={\left[{\left(29.5\right)}^{2}+0.357\text{Ar}\right]}^{1/2}-29.5$	[37]
Mass transfer coefficient	$K_{\text{sg}}=4.5(\frac{\beta e_{\text{mf}}}{d_{\text{pr}}})+5.85\left(\frac{PPC\cdot g^{4/1}}{{d_{\text{pr}}}^{5/4}}\right)$$K_{\text{gs}}=6.77\left(\frac{D_{g}0.45v_{b}}{{d_{\text{pr}}}^{3}}\right)$	[20]
Momentum exchange coefficient	$K_{\text{mn}}=150\frac{\alpha_{s}^{2}v_{g}}{\alpha_{g}d_{pr}}+1.75\frac{\alpha_{s}\rho_{g}}{d_{pr}}\left|v_{b}-v_{e}\right|$	[12]

#### 2.3.3. Mass Balance Model

The continuity equation for the gas and solid phases are as follows:

The continuity equation for the gas phase:

(10) $$\frac{\partial }{\partial t}\left(\alpha_{g}\rho_{g}\right)+\nabla \cdot \left(\alpha_{g}\rho_{g}{\vec{\nu }}_{g}\right)=\sum_{s=1}^{n}\left({\dot{m}}_{sg}-{\dot{m}}_{gs}\right)$$

The continuity equation for the solid phase:

(11) $$\frac{\partial }{\partial t}\left(\alpha_{s}\rho_{s}\right)+\nabla \cdot \left(\alpha_{s}\rho_{s}{\vec{\nu }}_{s}\right)=\sum_{g=1}^{n}\left({\dot{m}}_{gs}-{\dot{m}}_{sg}\right)$$

#### 2.3.4. Conservation of Momentum

The momentum balance for the gas phase

(12) $$\frac{\partial }{\partial t}\left(\alpha_{g}\rho_{g}{\vec{\nu }}_{g}\right)+\nabla \left(\alpha_{g}\rho_{g}{\vec{\nu }}_{g}{\vec{\nu }}_{g}\right)=-\alpha_{g}\nabla P+\nabla \cdot {\ddot{\tau }}_{g}+\alpha_{g}\rho_{g}\vec{g}+\sum_{s=1}^{n}\left({\vec{R}}_{sg}{\dot{m}}_{sg}{\vec{v}}_{sg}-{\dot{m}}_{gs}{\vec{v}}_{gs}\right)+\left({\vec{F}}_{g}+{\vec{F}}_{lt,g}+{\vec{F}}_{vr,g}\right)$$

where ${\ddot{\tau }}_{g}$ is considered as the specific gas phase stress-strain tensor and can be defined as

(13) $${\ddot{\tau }}_{g}=\alpha_{g}\mu_{g}\left(\nabla {\vec{v}}_{g}+\nabla {\vec{v}}_{g}^{T}\right)+\alpha_{g}\left(\lambda_{g}-\frac{2}{3}\mu_{g}\right)\nabla \cdot {\vec{v}}_{g}\ddot{Ι}$$

The momentum balance for the solid phase

(14) $$\frac{\partial }{\partial t}\left(\alpha_{s}\rho_{s}{\vec{\nu }}_{s}\right)+\nabla \left(\alpha_{s}\rho_{s}{\vec{\nu }}_{s}{\vec{\nu }}_{s}\right)=-\alpha_{s}\nabla P+\nabla \cdot {\ddot{\tau }}_{s}+\alpha_{s}\rho_{s}\vec{g}+\sum_{g=1}^{n}\left({\vec{R}}_{gs}{\dot{m}}_{gs}{\vec{v}}_{gs}-{\dot{m}}_{sg}{\vec{v}}_{sg}\right)+\left({\vec{F}}_{s}+{\vec{F}}_{lt,s}+{\vec{F}}_{vr,s}\right)$$

Stress-strain tensor solid phase:

(15) $${\ddot{\tau }}_{s}=\alpha_{s}\mu_{s}\left(\nabla {\vec{v}}_{s}+\nabla {\vec{v}}_{s}^{T}\right)+\alpha_{s}\left(\lambda_{s}-\frac{2}{3}\mu_{s}\right)\nabla \cdot {\vec{v}}_{s}\ddot{Ι}$$

The solid phase stresses were described according to the KTGF theory [42], where the random particle motion is modelled by analogy with the thermal motion of molecules in a gas using the concept of granular temperature.

The given solids’ granular temperature corresponds to the kinetic energy of the particles’ random motion. The equation below is derived from the kinetic theory for granular temperature:

(16) $$\frac{3}{2}\left[\frac{\partial }{\partial t}\left(\alpha_{s}\rho_{s}\Theta_{s}\right)+\nabla \cdot \left(\alpha_{s}\rho_{s}{\vec{\upsilon }}_{s}\Theta_{s}\right)\right]=\left(-p_{s}\ddot{Ι}+{\ddot{\tau }}_{s}\right):\nabla {\vec{\upsilon }}_{s}+(\nabla \left(k_{\Theta_{s}}\nabla \Theta_{s}\right))-{\lambda \Theta }_{s}+\phi_{gs}$$

where $-p_{s}\ddot{Ι}{\ddot{\tau }}_{s}$ = the generation of energy by the polypropylene particle stress tensor; $k_{\Theta_{s}}\nabla \Theta_{s}$ = diffusion of energy ($k_{\Theta_{s}}$ is the diffusion coefficient); $\lambda \Theta_{s}$ = collisional dissipation of energy; $\phi_{gs}$ = energy exchange between the certain point of gas phase and solid phase or *vice-versa*. Equation (16), comprises the term $\left(k_{\Theta_{s}}\nabla \Theta_{s}\right)$, relating the diffusive flux of granular energy. When the default Gidaspow *et al.* [43] model is enabled the ANSYS FLUENT uses the following expression

(17) $$k_{\Theta_{s}}=\frac{150\rho_{s}d_{s}\sqrt{\Theta \pi }}{384\left(1+e_{ss}\right)g_{o,ss}}{\left[(1+\frac{6}{5}\alpha_{s}g_{o,ss})\left(1+e_{s}\right)\right]}^{2}+2\rho_{s}{\alpha_{s}}^{2}d_{s}\left(1+e_{ss}\right)g_{o,ss}\sqrt{\frac{\Theta_{s}}{\pi }}$$

where, $e_{ss}$ refers to the restitution coefficient of the granulated solid particle (particle-particle), $g_{o,ss}$ refers to the radial distribution function and $\Theta_{s}$ represents the polymer’s granular temperature. ANSYS FLUENT is characterised by a 0.9 default. However, it can be tailored with accordance to the particle type. Several research groups [22,23,44] support the notion that the diffusive terms and the convection can be disregarded, given a local occurrence of the granular energy’s dissipation and its constant condition. Taking into account the complexity of the partial differential equation, overlong computational hours and instabilities in the solution method, the algebraic type of the equation has been suggested by many research groups for simulating fluidised beds [12,30,32]. Therefore, an algebraic equation can be derived to calculate the granular temperature on the basis of Equation (16).

(18) $$0=\left(-p_{s}\ddot{Ι}+{\ddot{\tau }}_{s}\right)\nabla {\vec{\upsilon }}_{s}-{\lambda \Theta }_{s}$$

The granular temperature can be wholly or partially computed using the options and preferences listed below:

- the default algebraic equation based on Equation (16), which disregards any diffusion and convection in transport;
- a partial equation of the differential based on Equation (16), which uses various property options;
- the constant value of the granular temperature which can be applied in the cases of small arbitrary variations;

#### 2.3.5. Solids Pressure

The total solid pressure was calculated and included in the mixture momentum equation:

(19) $$P_{\sum \text{solid}}=\sum_{s=1}^{N}p_{gr}$$

For the granular particulate flow in the fluidised bed regime, the solid pressure was calculated independently and used for the pressure gradient term, $\nabla p_{s}$ in the granular-phase momentum equation. The solid pressure was composed of a kinetic term and a second term due to particle collisions:

(20) $$p_{gr}=\alpha_{s}\rho_{s}\Theta_{s}+2\rho_{s}\left(1+e_{s^{\prime }}\right){\alpha^{2}}_{s}g_{0,s^{\prime }}\Theta_{s}$$

The value of $e_{s^{\prime }}$ in this study was set at 0.9, but the value can be adjusted according to the particle type. The granular temperature $\Theta_{s}$ is proportional to the kinetic energy of the fluctuating particle motion. The function $g_{0,s^{\prime }}$ is a distribution function that governs the transition from the minimum fluidisation velocity. The simulation criteria for the pilot scale fluidisation study generally suggest and advise that the gas velocity be varied from three to seven times that of the minimum fluidisation velocity [12,45]. Since the ANSYS FLUENT provides a default value of 0.63 for $g_{0,s^{\prime }}$ a minimum fluidisation value of 0.1 m/s was considered for the simulation in this study.

## 3. Results and Discussion

According to the design, 20 batch experiments were performed with various combinations of the process parameters. The propylene polymerisation percentage (*Y*_ppc_) was considered the response to the developed model. The design of the experiment on the process parameters under consideration and the achieved results are listed in Table 3.

**Table 3:** Experimental design and results of the response surface design.

Run	Factor A RT (°C)	Factor B SP (bar)	Factor CMC (%)	Response, Y_ppc_, (%) (Actual)
1	70	25	75	5.96
2	70	25	75	4.83
3	70	20	70	4.53
4	80	30	70	5.10
5	75	20	75	5.90
6	70	30	70	4.57
7	75	25	70	5.62
8	75	25	75	5.98
9	75	25	80	5.94
10	70	20	80	5.63
11	75	25	75	5.96
12	75	25	75	5.97
13	75	25	75	5.95
14	80	25	75	5.89
15	70	30	80	5.53
16	75	25	75	5.95
17	75	30	75	5.92
18	80	30	80	5.95
19	80	20	70	4.98
20	80	20	80	5.93

### 3.1. RSM Analysis

It is highly desirable to study the correlations between process variables and responses, and RSM is exceptionally well-suited for extensive chemical reactions comprising single or multiple responses [46,47]. The RSM-based quadratic model for the propylene conversion rate can be presented by Equation (21):

(21) $$\begin{array}{c} Y_{\text{ppc}}=\left(0.28\times A\right)+\left(0.002\times B\right)+\left(0.42\times C\right)+\left(0.025\times \text{AB}\right)-\left(0.032\times \text{AC}\right)-\left(0.030\times \text{BC}\right)\\ -0.55\times A^{2}+0.038\times B^{2}-0.13\times C^{2}+5.94 \end{array}$$

where *Y*_ppc_ is predicted monomer concentration and *A*, *B*, and *C*, are reaction temperature, system pressure, and monomer concentration respectively.

**Figure 3:**
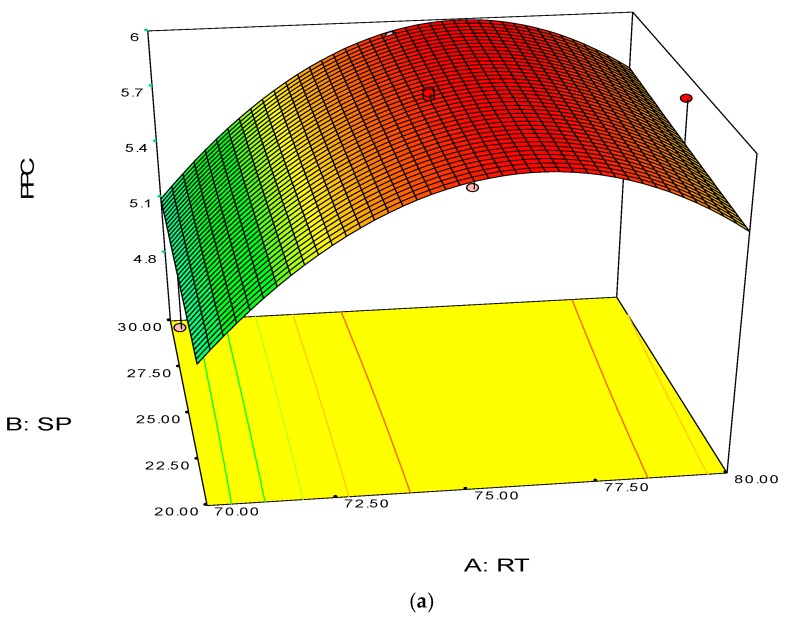

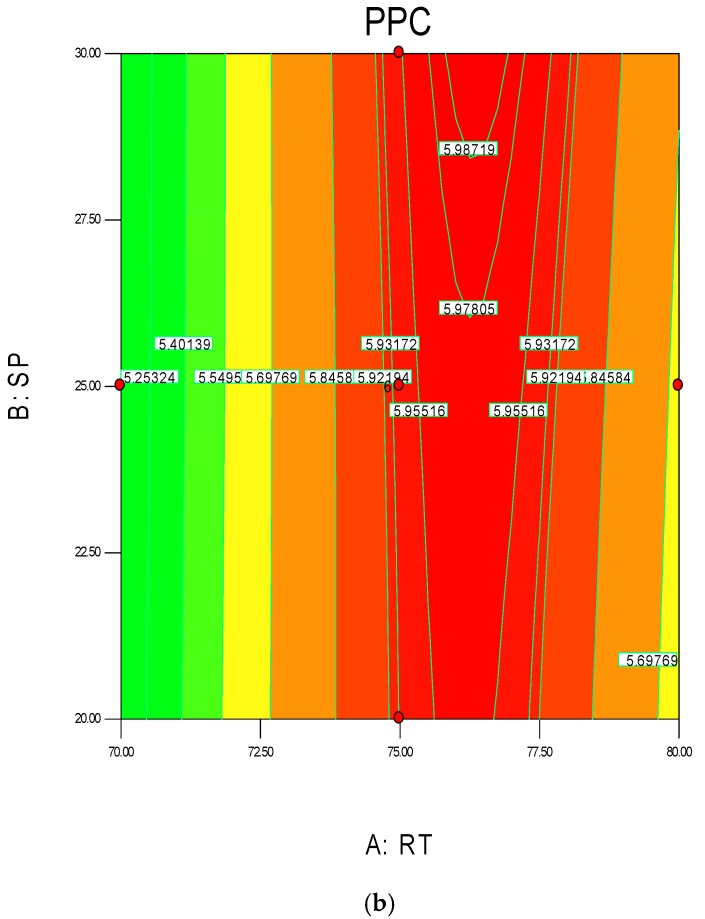
3D response surface (**a**) with 2D contour plot (**b**) of reaction temperature (RT) and system pressure (SP).

**Figure 4:**
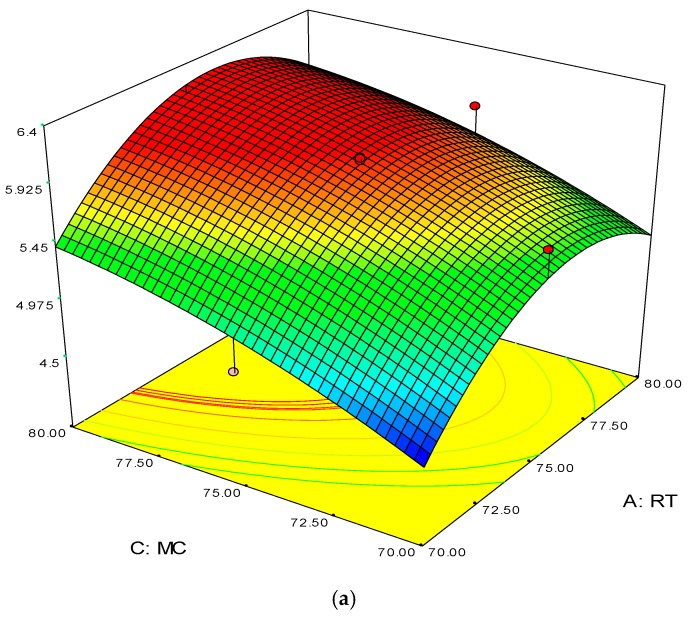

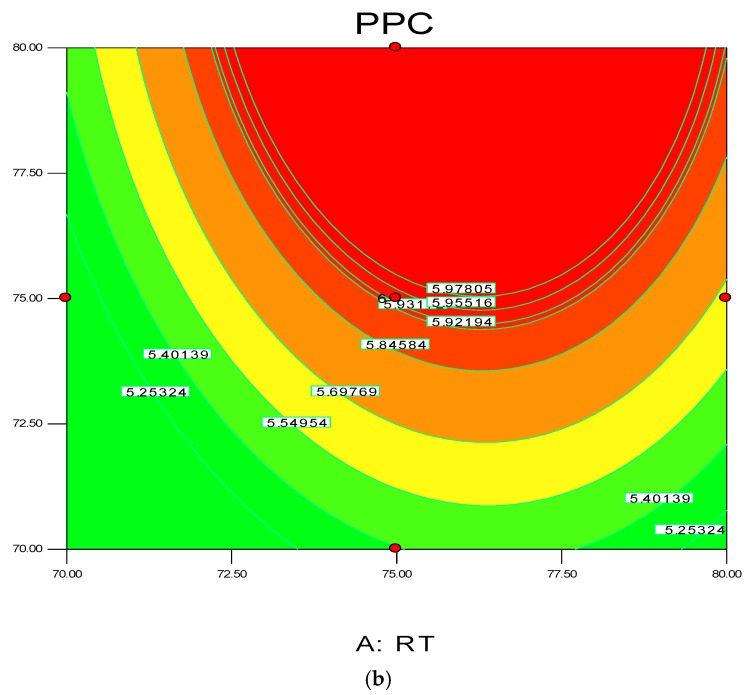
3D response surface (**a**) with 2D contour plot (**b**) of reaction temperature (RT) and monomer concentration (MC).

The 3D surface and 2D contour plots are shown in Figure 3 and Figure 4. The interaction structure of two process parameters can be explained by setting another fixed parameter at the central level by applying Equation (21). The 3D plot in conjunction with the contour investigation has also been employed to verify the optimum process parameters for the highest response of polymer conversion yield at the surfaces.

Each combined 3D and 2D figure signifies the optimum results of two independent process variables, where the blue to red coloured line signifies the lowest to highest response level ranges respectively. The highest response value was found on the area separated by the red coloured lines in the 3D and contour diagram. Figure 3 and Figure 4 direct the major interactions amid any two process parameters on the polymer conversion, when the other process parameter was fixed at their central points.

Figure 3a,b shows the effect of temperature and pressure on the polymer conversion rate. The conversion rate showed a rising trend with increments of reaction temperature and system pressure, up to a certain level. From the response plot, the increment of response values can be clearly seen. At a temperature of 75 °C the response point value is 5.98%, and when the temperature increased to 77.5 °C, it gave the same response value. Further increments in temperature showed a decrease of response. The conclusion can be drawn that the optimum temperature is 75 °C. However, most of the optimum responses values are at the 25 bar point, noticeable from the contour plots. At 25 bar the propylene polymerisation percentage response value remains at about 5.93%–5.98%. The increase in pressure above 25 bar does not show any significant improvement in the response value, whereas the optimum zone starts at 25 bar. In the literature, system pressure fluctuation has been described as an important parameter for olefin polymerisation with the fluidisation technique, as it can affect the bed dynamics [48]. Optimum fluidisation yield was studied by researchers at pressure ranges of 1–16 bar [49]. Experimental results reported a substantial boost in the total fluidisation performance with pressure increases up to 15 bar [50]. However, it is noteworthy that the reports were derived from lab-scale virtual analysis and were not results from real reaction conditions, and a minimum pressure of 20 bar is mandatory to produce industrial grade polypropylene [26].

The 3D surface plots in Figure 4a,b show the propylene conversion rate sharply increases due to increments in reaction temperature and monomer concentration. However, a consistent rise of monomer concentration also illustrates significant changes in the propylene conversion rate. The optimum response value was obtained at 75% MC and further increments did not show any notable changes on response. Therefore, it is concluded that at 75% MC the response value 5.98% is achieved. From the literature, seen through purely mathematical modelling, it is evident better polypropylene production rates can be achieved at the emulsion phase with consistent increments in temperature and propylene concentration [20]. Some studies [26,27] showed that at higher emulsion phase temperatures and lower monomer concentrations, the propylene yield was unchanged, which indicates to a certain extent variation of the monomer concentration does not affect the production rate. The finding strongly supports the result of this study.

**Figure 5:**
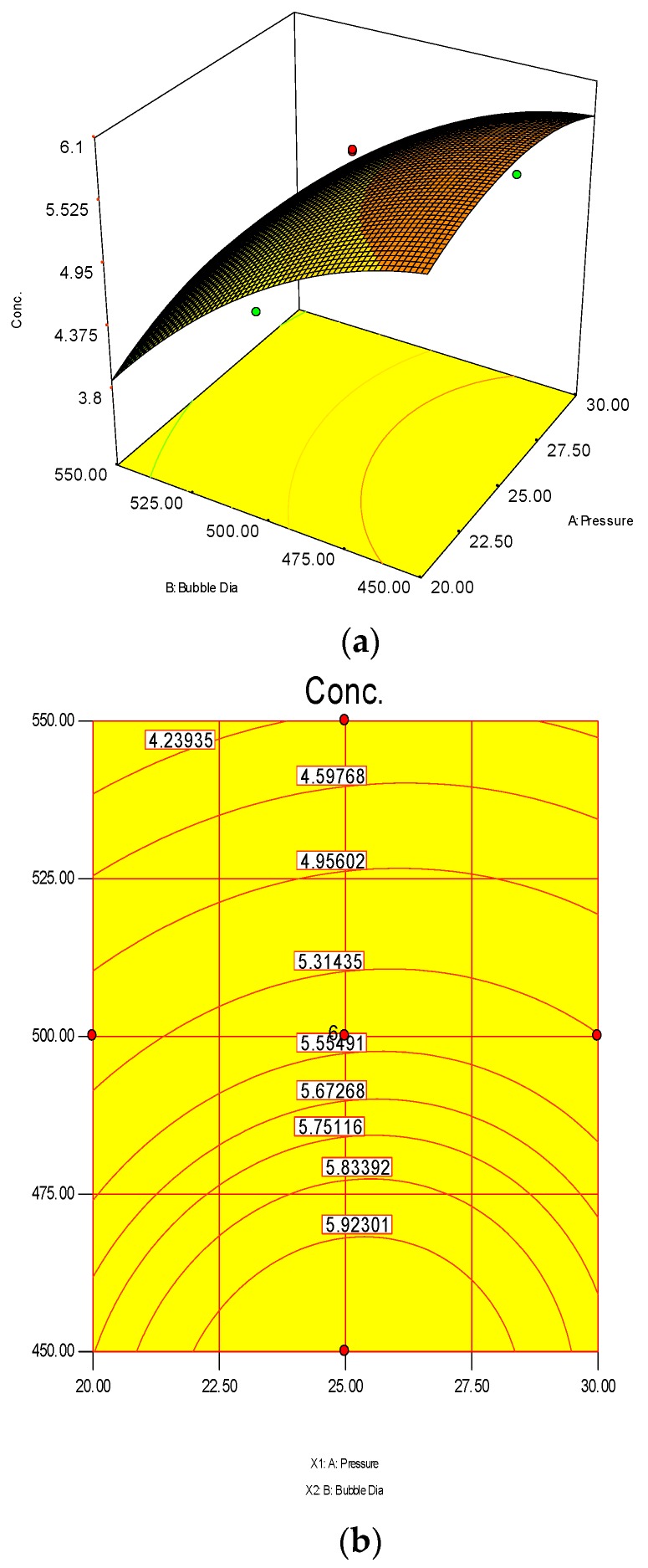
3D response surface (**a**) with 2D contour plot (**b**) of bubble diameter and pressure on polymerisation.

A thorough analysis of previous literature regarding gas-phase propylene polymerisation models has indicated that the topic of significant polymerisation examined through the bubble size effect has been neglected so far. According to Shamiri *et al.* (2010) [27], however, this catalyst action during the bubble phase is to be considered mandatory when building a model. Both the emulsion and bubble phases witness polymerisation reactions due to the fact that the bubbles also include solids. Figure 5 shows estimated total propylene polymerisation with regard to the bubble size and system pressure in the bubble phase. This is so because fluidisation is expected to lead to variation in the bubble size. The diameter of the bubble can vary between 4.50 × 10^−4^ and 5.50 × 10^−4^ m. This formula demonstrates that the smaller the bubble size, the higher the polymerisation percent will be. On the other hand, the highest value of the bubble size (4.50 × 10^−4^ m) along with bar pressure of 25 results in the highest rate of polymerisation (5.92%/pass).

### 3.2. Effect of Process Conditions on Bed Structure during Reaction

In this section, the hydrodynamic features under specific operating conditions are explored. The current study adopted a fluidised bed reactor from the simulated gas phase, which is identical with the propylene polymerisation reactor in its pilot scale used by the University of Malaya’s Department of Chemical Engineering. The fundamental reason for this setup adoption is to examine operating conditions comparable to the ones in the industrial units, as well as olefins’ catalytic polymerisation when subjected to high pressure. Section 2.1 elaborates on the pilot plant’s specific characteristics. The medium of the fluidisation contains a monomer gas combination of hydrogen, nitrogen, also called inert gas, and propylene.

#### 3.2.1. Boundary Conditions

The uniform inlet velocity was conceived as an inlet boundary condition, while the top of the bed took the form of a fixed pressure outlet. Table 4 depicts the thorough plan summary of the fluidised bed reactor’s CFD simulation pilot scale. The functional superficial gas velocity was set between 0.5 and 0.75 m/s in the pilot plant, which accounts for its cylindrical geometry. The superficial gas velocity dimension was thus evaluated in terms of rectangular geometry due to the overwhelming calculation expenses, in order to coordinate the accessible plant information with the height of the bed and process parameters. Last but not least, existing literature has deemed 0.5 m/s an appropriate gas velocity value, thus it has been assigned to the experiment. Front and back walls aftermath has been disregarded. The gas phase was assigned no slip wall boundary conditions, while the solid phase was given the free-slip ones. For the cases when there is no solid phase, a uniform gas inlet velocity was induced by applying the Dirichlet boundary condition. At *t* equalling zero, all velocities were also assigned zero value. The bed’s assumed condition was the initial well-mixed one, while the condition of the outlet pressure boundary was given a 25-bar value. The current study operates with one gas phase and three particle ones (quadrature points). The primary phase was assumed to be the gas phase. The particle phases, involving polymer particles, were distinguished by multiple properties, such as volume fraction, particle shape factor, length, density, *etc.*; the quadrature weights and the variations of the weighted nodes have been nullified. Particle density was assumed to be 910 kg/m^3^, and the viscosity and inlet gas densities as 1.14 × 10^−^^4^ Pa·s and 23.45 kg/m^3^. The values were set to match a pilot scale gas-phase polymerisation reactor’s characteristics. The packing fraction was assigned a maximum value of 0.75 because the space surrounding the larger particles was presumed to be filled by the smaller ones. The coefficient of the restitution was set to 0.8. Another important inference was that the heat emitted during the reaction was thoroughly removed and the bed was able to maintain an isothermal condition [16]. Table 4 presents the simulation and wall boundary conditions accordingly.

**Table 4:** Boundary conditions for simulation set ups.

Factors		Value
Reaction zone	Inner diameter	0.1016 m
	Cross sectional area	0.00785 m^2^
	Height	1.5 m
	Volume	0.011775 m^3^
Disengagement zone	Inner diameter	0.25 m
	Cross sectional area	0.0490625 m^2^
	Height	0.25 m
	Volume	0.0097 m^3^
Reactor volume		0.0215 m^3^
Initial bed height (m)		1.5
Initial void fraction		0.431
Gas density (kg/m^3^)		23.45
Gas viscosity (Pa·s)		1.14 × 10^−^^4^
Particle density (kg/m^3^)		910
Coefficient of restitution		0.8
Angle of internal fraction		30
Maximum solid packing volume fraction		0.75
Time step (s)		0.001
Activation energy, *E* (J·mol^−^^1^)		7.04 × 10^4^
Active site of catalyst (mol·m^−^^3^)		1.88 × 10^−^^4^
Feed monomer concentration (mol·m^−^^3^)		0.75
Pre-exponential factor, *kp*0 (m^3^·mol^−^^1^·s^−^^1^)		1.2 × 10^4^

#### 3.2.2. Model Validation and Grid Sensitivity Analysis

Model validation required time step and grid sensitivity analysis, executed by correlating the information from the pilot scale gas-phase polymerisation reactor and the results from the simulation. Table 3 and Table 4 illustrate the conditions of the simulation. The phase formation event determines the median bed high on the basis of its catalyst properties and injection, product separation devices and withdrawal position, particle residence time, and operating condition. Hence, affirmation purposes appealed for propylene conversion inside the reactor. Variations in the bed height are determined by changes in the process parameters, which represent vital fluidisation attributes like bubble hydrodynamic, the bed’s operating conditions, and gas turbulence. The bed height and the pressure drop’s transient behaviour are compared to the data acquired by the pilot plant, as displayed in Figure 7. It is evident that the simulation course comprises start-up and quasi-steady fluidisation stages. Pressure drop oscillation most often occurs within the operational range, which is caused by the attributes of the fluidisation, while the gas-solid flow can witness a steady state of the bed height after 73 s.

#### 3.2.3. Grid Independent Analysis

**Figure 6:**
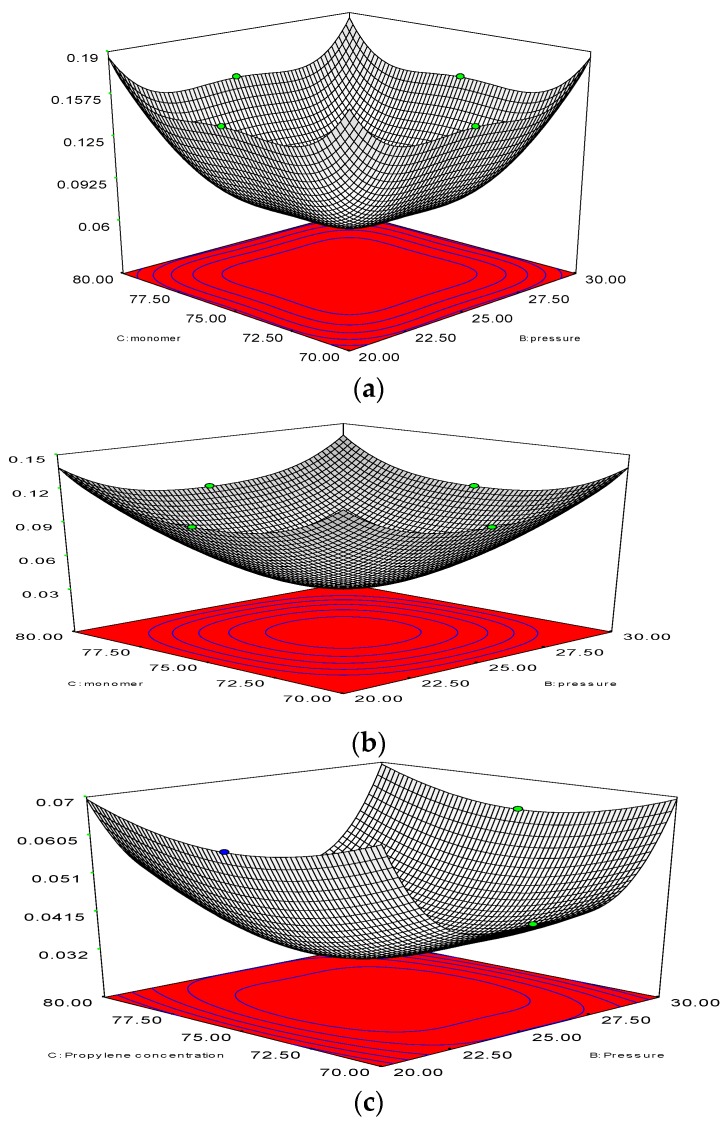
(**a**) Changes of polymerisation rate at node number 101,343 at various pressure and amount of monomer (propylene). *y*-Axis indicates the polymerisation changes; (**b**) Changes of polymerisation rate at node number 101,343 at various pressure and amount of monomer (propylene). *y*-Axis indicates the polymerisation changes; (**c**) Changes of polymerisation rate at node number 87,009, at various pressure and amount of monomer (propylene). *y*-Axis indicates the polymerisation changes.

With the help of a 2D analysis and a boundary-and-gradient adaptation technique, it was confirmed that the higher the resolution the more independent of grid the outcome is. In this way, the adjoined mesh points could be situated in high-gradient areas in the inlet and fluidisation regions. The response variations at three mesh resolutions with 50,464, 87,009, and 101,343 node numbers correspond to Figure 6a–c. The parameters for the simulation include 1.5 m of bed height, 1000 s real time, and 0.5 m/s superficial gas velocity. Figure 6 illustrates the three separate grids, which were used to partition the 2D flow domain into square cells.

**Figure 7:**
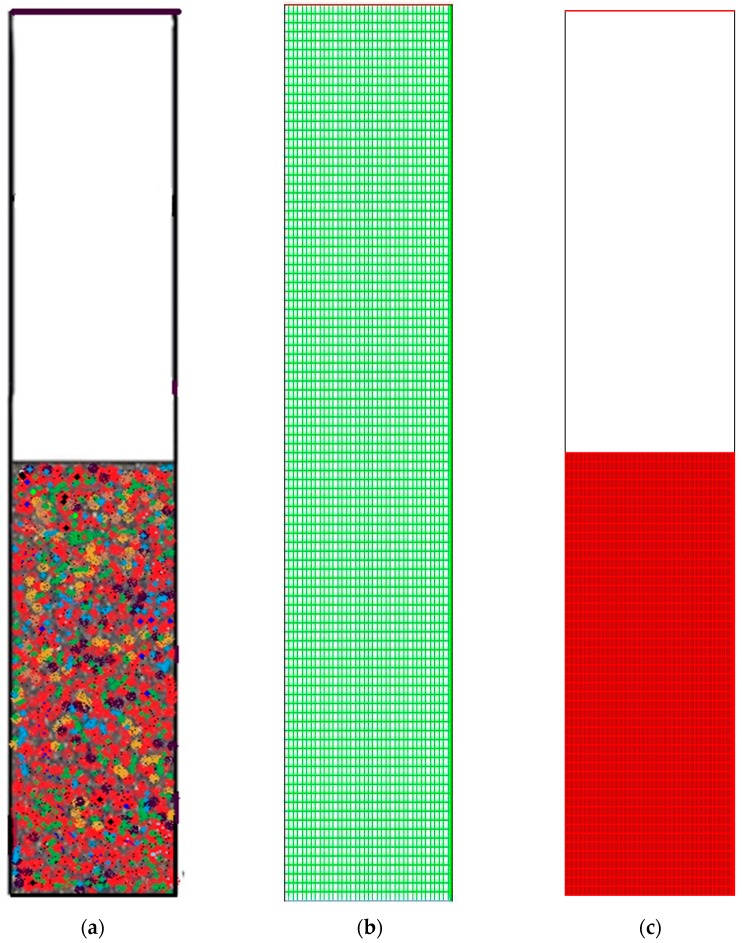
General computational domain and mesh generation. (**a**) Framework of the gas-phase fluidised bed polymerisation reactor used in simulation; (**b**) Generated Mesh for fluidised bed simulation; (**c**) Computational region marked.

It is evident from Figure 6a–c that grid resolution plays a determinative role for the response. Thus, it can be deduced that the polymerisation variation is in the 0.07%–0.14% range, according to the nodes variation, which ranges from 87,009 to 50,464. Nonetheless, with the upsurge of the grid resolution (from node number 87,009 to 101,343), the response value also increases in the 0.14%–0.19% range. Hence, the smaller the variation, the more accurate the response calculation will be. What is preliminary in this scenario is for a compromise to be established between the time for calculation and the necessary accuracy. As a result, adequate grid convergence with a minute polymerisation difference of 0.07% at 87009 nodes is needed to attain more precise results during the simulation on the pilot scale. However, overall computational domain and mesh generation has been depicted in Figure 7. A sketch of the fluidised bed filled with granulated particles is shown in Figure 7a. Meshing and the marked domain are given in Figure 7b,c.

#### 3.2.4. Fluidized Bed Dynamics at Various Set of Process Conditions

**Figure 8:**
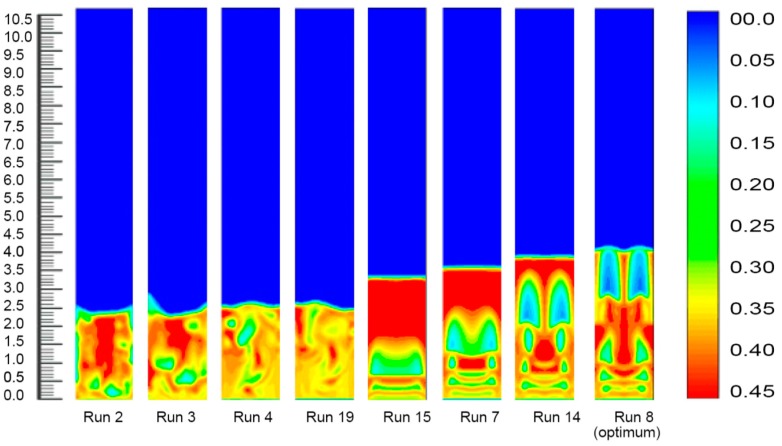
Bed dynamics at different set process combinations.

Fourteen sets of process conditions were selected for the simulation study as this combined a set of eight process parameters which covered the remarkable range of polymer conversion percentages. The inlet gas mixture velocity was fixed at 0.63 m/s in the simulations, and the corresponding simulated results are shown in Figure 8.

Figure 8 depicts the solid volume fraction profiles at different fixed gas velocities and time under diverse operating conditions. As clearly indicated in the figures, an alteration in the temperature, pressure and propylene concentration led to a rising trend in the bed expansion height and an increase in the bubble size. It was also obvious that probable negative deviations were noticed over the common parameter space, but strong positive variations were detected for variations in the system pressure, which also led to developments in the bed heights. This suggests that within these ranges the simulations, that were exclusive of the presence of variations of pressures, forecasted better reactor performance (higher degree of polymerisation) even with the much denser (and lower penetrable) emulsion phase. The cause of this effect was best portrayed by animations of the volumetric segment of the two phases, which can also be seen in the figures. Fundamentally, the alteration in the system pressure expressively raised the solid phase trajectories, instigating the bed to act more solid-like to a certain degree. This initiated the appearance of bigger sized bubbles at the reactor inlet point and the construction of consistent channelling for the fluid (gas) through the bed. Individually, these trends reduced the characteristics of the gas–solid contact and hence, lessened the reactor performance. Conversely, however, if the system pressure increased to a certain level (from 20 to 25 bar), the bed acted very liquid-like. Small bubbles were formed at the inlet and less channelling was observed. The most likely reason is that with the alteration of the bed, the thermo-physical vectors affected the particle movement in the bed sharply and assured more uniform contact between the gas, solid, and catalyst. This would have resulted in an increase in the inter-particle forces (including the drag force) between the gas and solid phases acting on the particles. At a lower system pressure, the particles accumulated in the lower portion of the bed. As the gas pressure increased, the solid volume fraction at the bottom of the bed increased gradually. Thus, the bubble size and the bed expansion height apparently increased. The systematic bubble development and movement are very important for the expected mixing of the solid and the gas, which also ensures the achievement of a better polymer conversion rate [51]. On the other hand, Figure 5 illustrates the bed condition at a comparatively lower pressure (20 bar) and temperature (70 °C), and expresses a comparatively mediocre bubble orientation. At this set of operating conditions (Run No. 2) the rate of propylene conversion was also lower. As depicted in Figure 8, the solid volume fractions became uniformly distributed in the core region across the bed, and significant differences were found at the upper region of the reactor. This means that after the gas had carried the granules to the top of the bed, they were jetted out and the polymer particles were circulated back down along the bubbles for the impact of the bed expanding section. The comparison and analysis of the hydrodynamic characteristics in Figure 8 show that when the pressure and temperature were at optimum conditions (run 8) in the bed, the bubble formation and movement, which are responsible for imparting the gas–solid contact, were changed remarkably. The wide-ranging contact is responsible for higher polypropylene production in real conditions [31].

### 3.3. Examining the Model Accuracy

Varying in the response value can take place if the factor level is altered by coding a particular unit, represented by the coefficient of the developed equation. Analysis of variance and *F*-value were considered to examine the equation model and the consequence of second-order models at 95% confidence level. In practice, a larger calculated *F*-value than tabulated *F*-value suggests the null hypothesis should be avoided, as the values of individual regression coefficients trend to zero. The *F*-value can be formulated by the following equation:

(22) $$F=\frac{MnS_{\text{RG}}}{MnS_{\text{RD}}}$$

**Figure 9:**
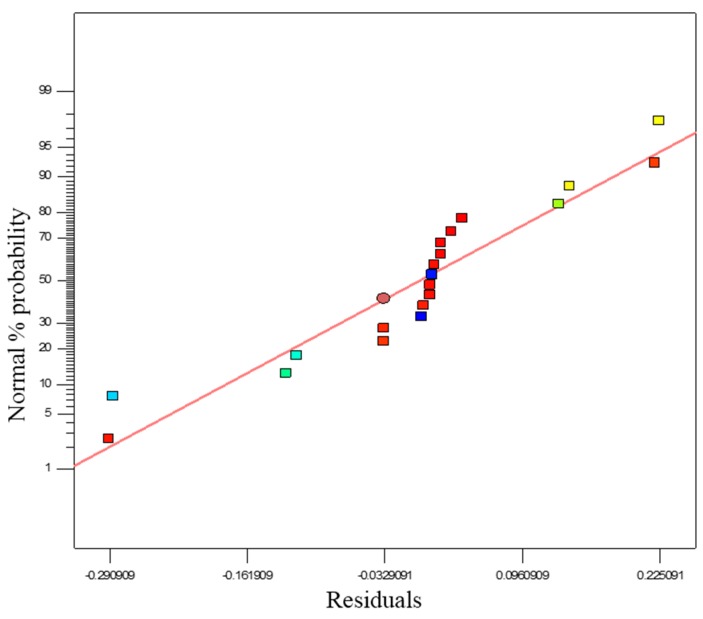
Normal % probability and residual plot for propylene polymerisation (%).

ANOVA findings were used to check model accuracy, together with other significant statistical diagnostic tools. Normal probability and residuals plots for the propylene conversion rate are shown in Figure 9. The normal probability test evaluates the data set applied in the model and whether or not it is normally distributed. According to normal distribution theory, the plotted data should be compared to a projected straight line. Any divergence of the plotted data from the projected line would signify a digression from normality. If a linear shape is formed from the plotted data, it can be concluded that the data is distributed normally. In Figure 9, the fit of the model data and of the degree of concurrence with the results of the ANOVA are shown, where the residuals calculate the quantity of standard deviations in both experimental and predicted values. Figure 9 also suggests that response transformation analysis can be avoided as no further perceptible problem is found with normality.

**Figure 10:**
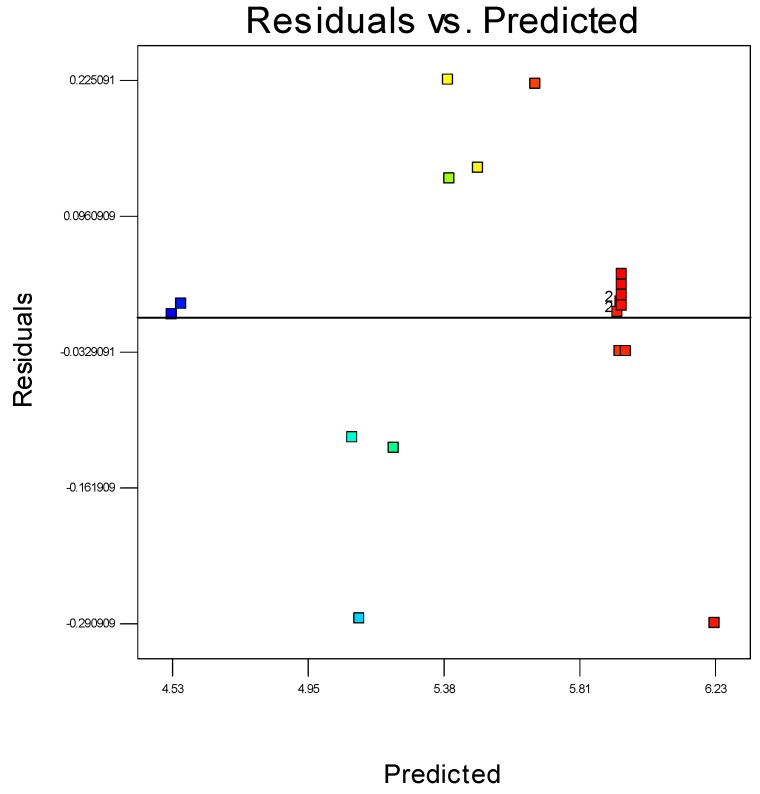
The residuals and predicted response plot for propylene polymerisation.

Residuals are considered as estimations of experimental error, attained by deducting the actual from the estimated response. Theoretically, the estimated response can be determined from the selected model, as the model parameters are assessed from experimental data. Precise investigation on residuals can express whether the hypotheses are satisfactory and the model selection is suitable. In a regression model it is expected that the error should appear randomised. The conclusion would be if the estimates of the model are greater than the actual values, but lesser than the actual with identical probability. Furthermore, the range of the error must also be independent otherwise the scope of the observation may remain predicted. It could be expected that the pattern of the residuals would have a scattered form. Accordingly, graphical methods are important to observe residuals. A randomly scattered plot of residual and predicted values can be seen in Figure 10. The collective impression is that as the plot is randomly scattered, the variance of real observations is stable for each response value. This also suggests there is no need for transformation of the response variable.

**Figure 11:**
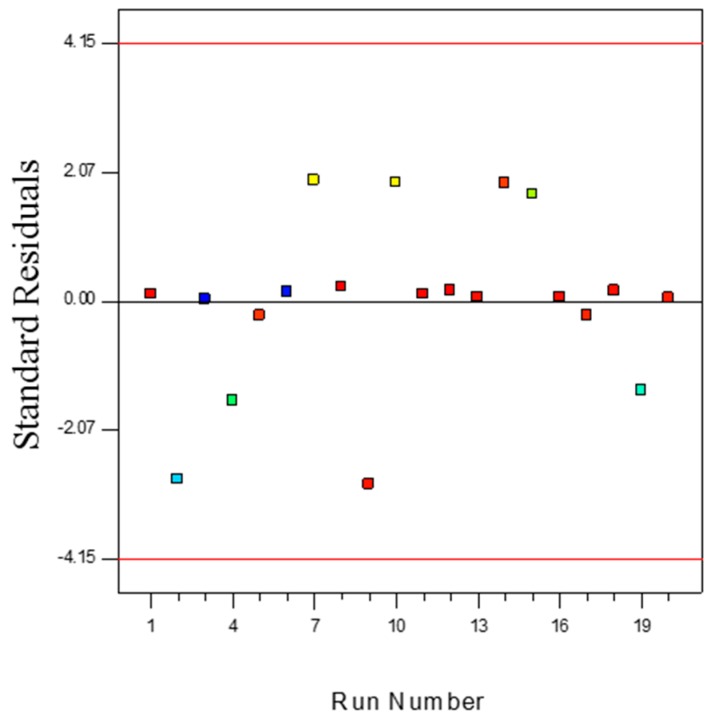
Outlier *t* plot for propylene polymerisation (%) per pass.

The measurement of the number of deviation points of experimental values from predicted values is an important step for statistical digenesis of an experimental design. The outlier *t* measurement can provide a clear explanation on this matter. Figure 11 illustrates the outlier *t* plot for propylene polymerisation (%) over the batch reactions carried out. All the standard residuals positioned between ±3.50 suggest the estimation of the fitted model towards the response surface was positive, which also suggests data recording errors are negligible. However, any data that falls outside this range indicates the presence of insignificant terms in the model, and further investigation of the nonlinear influence of the specific parameters on response is required. In this type of situation, certain combinations of process parameters need to be repeated.

#### 3.3.1. Interaction Graphs

**Figure 12:**
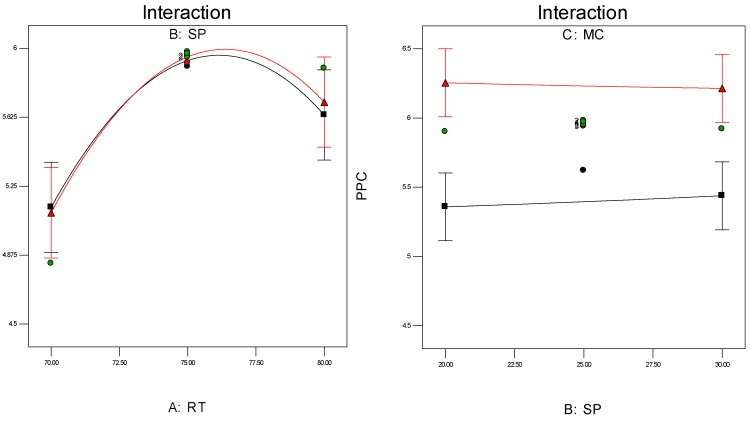

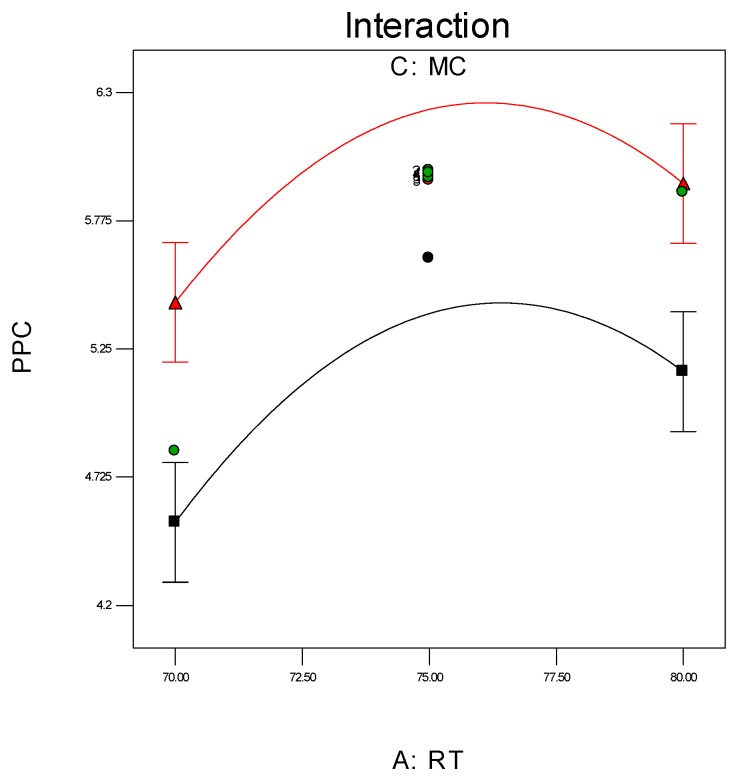
(**a**) Interaction between temperature and pressure; (**b**) Interaction between temperature and propylene concentrations; (**c**) Interaction between pressure and propylene concentration.

Investigation of the interaction effect among the process variables is essential to make decisions on optimisation in any chemical process. The RSM offers a convenient approach to monitor this issue, as it can clearly characterise the effects of binary arrangement by relating two independent variables. Interaction takes place once a specific factor does not generate the identical effect on the response at discrete levels of a new factor. So, if the graph curves of two factors are running parallel, there is no possibility for interaction to take place. If the interaction graph displays non-parallel curves, it indicates a relatively robust interaction between the process variables. Figure 12a,c, confirms the process variable interaction for each of the responses. Figure 12a shows a strong interaction effect between reaction temperature and system pressure, where the effects of binary combination of two independent factors can be easily recognised. However, Figure 12b,c does not show any non-parallel curves, signifying there was simply no interaction possible during the propylene polymerisation reactions.

#### 3.3.2. Perturbation Graph

**Figure 13:**
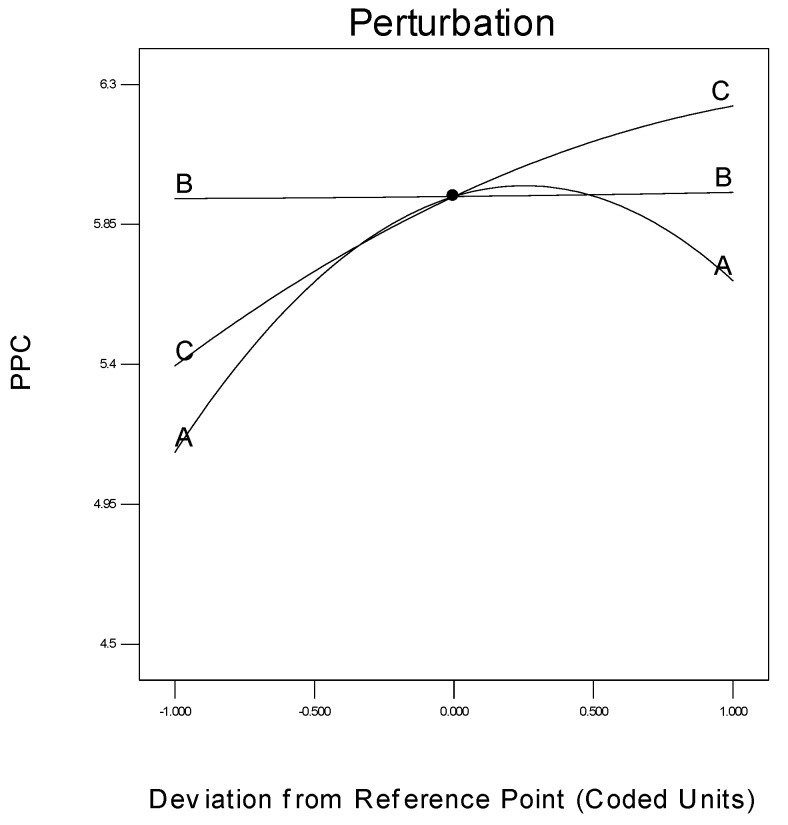
Deviation of individual parameter from the response.

The specific effect of every parameter on the response is another important concern in process modelling, which can be shown by a statistical measure termed a perturbation plot. This plot facilitates the comparison of the influences of every process parameter based on the centre point inside the design plot. Figure 13 is the perturbation chart for the polymer conversion rate with respect to A, B, and C. The perturbation plot signifies the effect of a certain parameter at a specific designed point of extent. The response, *i.e.*, the polymer conversion rate (in percentage) of propylene is plotted by altering just one process parameter at a time over its extent, while maintaining the two other process parameters constant, at its centre point.

A perturbation plot shows the comparative influences of every independent process parameter on the polymer conversion rate. In Figure 13, a sharply bending curve in temperature (A) and monomer concentration (C), confirm that the response polymer conversion productivity was identically sensitive to these two process variables. Comparatively, the semi-flat system pressure curve (B) displays less sensitivity to alter the response efficiency, in respect of a change in propylene concentration. In other words, the monomer concentration has no major function in the polymerisation process, when comparing reaction temperature and pressure.

### 3.4. Statistical Diagnosis of the Model through ANNOVA Analysis

To analyse the most imperative effects and interactions, ANOVA analysis was applied and the results are given in Table 5. The *F*-value of the model at 14.80 specifies the significance of the model, and there is a negligible chance of error due to noise being present. Smaller Prob >*F* values (less than 0.05%) are a powerful indicator of the significance of model variables. Values greater than 0.1000 determine the model variables are non-significant. In this study, reaction temperature and monomer concentration are significant model variables. As the Prob > *F* values for RT and MC are 0.008 and <0.001 respectively, it gives the idea that the response can be severely affected when the reaction temperature range fluctuates and the monomer concentration is not properly controlled within a specific range.

**Table 5:** Statistical parameters for developed model and process parameters.

Functions	Degree of freedom,df	Mean Square	*F*-Value	*p*-Value (Prob > *F*)
Model	9	0.57	14.80	<0.0001
A-RT	1	0.76	22.50	0.0008
B-SP	1	0.003	0.006	0.867
C-MC	1	1.75	51.62	<0.0001
A^2^	1	0.82	24.29	0.0006
B^2^	1	0.009	0.018	<0.9777
C^2^	1	0.044	1.31	<0.2796

*R*^2^, 0.9302; Adj. *R*^2^_adj_, 0.8673; Adequate Precision: 13.091.

In statistical modelling *R*^2^ is considered as one of the measures which results in the reduction of variability of the response. In spite of this, a greater *R*^2^ value does not suggest a better fit for a regression model. Adding more variables increases the *R*^2^ value without considering the statistical significance. The value of *R*^2^ lays fractionally between 0.0 and 1.0 without units and can be determined by Equation (1). Achieving higher values indicates a better fit of the model to the data set. The *R*^2^ value of the model is 0.9302 which is very close to 1, thus it can be agreed the developed model comprises the best fit data.

The term adjusted *R*^2^ (*R*^2^_adj_) is applied for the purpose of adjustment of the number of terms in the model. If the addition of model terms does not add any value, then the *R*^2^_adj_ value is lower than regular *R*^2^. In other words, if *R*^2^_adj_ is less than regular *R*^2^, it already indicates there is no necessity to add extra terms in the model. In this study, *R*^2^_adj_ is 0.8673, which suggests that the model does not need to consider any additional terms.

Principally, adequate precision is a measure of the signal to noise ratio. This statistical tool can provide information about factors by which the model can be judged by examining if it is adequate to navigate amid the design space, along with being capable to predict the response. The desired value of adequate precision is more than 4.0. In this case, the value of adequate precision gained is 13.091. This was defined by the following equation:

(23) $$\frac{\max(Y_{p})-\min(Y_{p})}{\sqrt{\frac{p\omega^{2}}{n}}}\geq 4$$

## 4. Financial Benefits

As mentioned previously, PETRONAS Malaysia (one of the biggest petrochemical hubs for national and multinational players, such as BASF, Reliance, Kaneka, Eastman, and Polyplastics) is the industrial collaboration partner of this research project and the pilot scale reactor is the prototype of the industrial scale reactor. The capacity of this plant is 80,000 TPA (tonnes per *annum*) of polypropylene production through gas phase catalytic technology [52]. In fact, it is predicted that a 5.98% increase in production, from this advanced research, will generate extra profits of over €5.194 million per annum (at a cost of €1,197/metric ton) for this single polyolefin plant in Malaysia. However, a market research has predicted that the global demand for polypropylene will grow to 102 million TPA in 2016 [53]. From this estimation, the additional 6,411,981.74 TPA of polypropylene can be produced to meet this global demand; which may generate extra profits in the global market, to reach more than €7,675.14 million in 2016.

## 5. Conclusions

The process parameters of the optimisation phenomenon in a fluidised bed reactor were investigated and are associated with the prediction of reaction temperature, system pressure, as well as monomer concentration. As gas phase catalytic fluidisation is a complex and exothermic reaction, the polymer production rate and product quality is highly affected by temperature, availability of an appropriate quantity of monomer, and fluctuations in reactor pressure. Therefore, all of these process parameters are imperative when designing and constructing a fluidised bed reactor. These values need to be controlled as accurately as possible from an engineering point of view. The optimal polymerisation was achieved at 5.98% per pass at a reaction temperature of 75 °C, a system pressure of 25 bar, and with a controlled monomer concentration of 75%. The literature reports a 3%–4% polymer conversion per reaction pass, by applying fluidisation technology. Therefore, the findings of this study may be extremely helpful to decision making, not only in the polyolefin sector, but also in opening new doors of research in the overall petrochemical industry. Analysis, using the response surface methodology in conjunction with central composite design, was used to model the influence of three process parameters on propylene polymerisation. Mathematical model equations were derived for the single response by using sets of experimental data and ANOVA. The normal probability test, residual test, and outlier *t* plots, showed the developed model had a significant fit with the experimental outcomes. However, the interaction graphs clearly depicted that only reaction temperature and system pressure show trends to interact with each other. Conversely, the perturbation test showed that reaction temperature and monomer concentration had a very sharp effect on polymerisation. One of the unique findings from this study is the bed structure changes in the course of polymer conversion changes. However, system pressure variation did not affect the production rate significantly. Therefore, the model and its correlated findings can be efficiently exercised within the design space, together with an excellent correlation coefficient with a 95% confidence level, on the design and suitable parameters of a fluidised bed reactor system.

## Supplementary Materials

Supplementary materials can be found at www.mdpi.com/2073-4360/8/2/47/s1. Figure S1: Globally unique pilot scale fluidised bed catalytic reactor schematic for propylene polymerization, designed and fabricated at Department of Chemical Engineering, University of Malaya, Malaysia.

## Notations

*A*	Cross-sectional area of the reactor (m^2^)
Ar	Archimedes number
ANOVA	Analysis of variance
*DgF*_mod_	Degree of freedom of mode
*DgF*_residual_	Degree of freedom of residual
*F*-value	Model significance
*H*	Bed height (m)
*MnS*_er_	Mean Square Error
*MnS*_RD_	Mean of square residual
*MnS*_RG_	Mean of square regression
*N*	Number of experiments
*P*	Number of model parameters
*p*	Pressure shared by all phases
*P*_mw_	Propylene molecular weight (kg/kmol)
*PPC*	Polypropylene Concentration
*P*_rt_	Rate of propylene consumption
*R*^2^	Determination coefficient
*R*^2^_adj_	Adjusted coefficient of determination
SD	Standard deviation
S_RD_	Sum of residual
S_RG_	Sum of squares
SSQ	Sum of squares
SSQ_mod_	Sum of squares of model
SSQ_residual_	Sum of squares of residual
*v*_0_	Superficial gas velocity (m/s)
*v*_mf_	Minimum fluidisation velocity (m/s)
*Y*_p_	Predicted value
*Y*_R_	Response yield
*Ε*	Error vector
ω^2^	Residual value
*d*_b_	Bubble diameter (m)
$\chi_{\text{mf}}$	Void fraction of the bed at minimum fluidisation
${\vec{\nu }}_{\text{g}}$	Velocity of gas phase
${\dot{m}}_{\text{sg}}$	Mass transfer from the solid to gas phase
${\dot{m}}_{\text{gs}}$	Mass transfer from the gas to solid phase
${\vec{\nu }}_{\text{s}}$	Velocity of solid phase
$\mu_{\text{g}}$	Shear viscosity of gas phase
$\lambda_{\text{g}}$	Bulk viscosity of gas phase
${\vec{F}}_{\text{g}}$	External body force for gases
${\vec{F}}_{\text{lt},\text{g}}$	Lift force for gas phase
${\vec{F}}_{\text{vr},\text{g}}$	Virtual mass force for gas phase
${\vec{R}}_{\text{sg}}$	Interaction force between solid–gas phases
${\vec{v}}_{\text{sg}}$	Interphase solid to gas velocity
