# Supplementary material for: Developed Hybrid Model for Propylene Polymerisation at Optimum Reaction Conditions
Source: Polymers (Basel). 2016 Feb 10;8(2):47. doi: 10.3390/polym8020047 (PMC6432575; doi:10.3390/polym8020047)
Supplement: Supplementary file 1 [file polymers-08-00047-s001.pdf]

Supplementary Materials: Developed Hybrid Model for Propylene Polymerisation at Optimum Reaction Conditions

Mohammad Jakir Hossain Khan, Mohd Azlan Hussain and Iqbal Mohammed Mujtaba

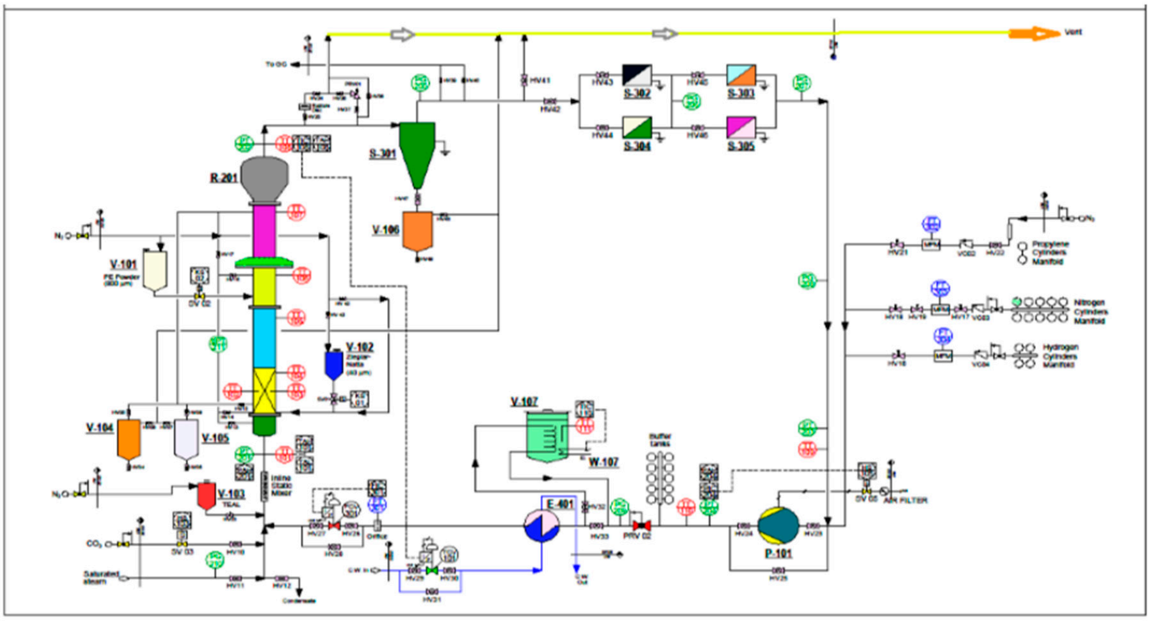

V-101<br>PP Powder Feed Tank<br>Stain less steel 304<br>0.20 m(D)×0.35 m(H)<br>Capacity Approx. 10-L	V-102<br>Catalyst Feed Tank<br>Stain less steel 304<br>0.10 m(D)×0.25 m (H)<br>Capacity Approx. 2-L	V-103<br>CO-Catalyst Feed Tank<br>Stain less steel 304<br>0.10 m(D)×0.15 m(H)<br>Capacity Approx. 500mL	V-103<br>CO-Catalyst Feed Tank<br>Stain less steel 304<br>0.10 m(D)×0.15 m(H)<br>Capacity Approx. 10-L	V-104<br>Product discharge Tank 1<br>Stain less steel 304<br>0.20 m(D)×0.35 m(H)<br>Capacity Approx. 10-L	V-105<br>Product discharge Tank 2<br>Stain less steel 304<br>0.20 m(D)×0.35 m(H)<br>Capacity Approx. 10-L	V-106<br>Cyclone Hooper<br>Stain less steel 304<br>0.15m(D)×0.15 m(H)<br>Capacity Approx. 2.5-L	V-107<br>Thermal Oil Bath<br>Heater 4kW<br>Capacity Approx. 20-L
R-201<br>Fluidized Bed Reactor<br>Stain less steel 304<br>4″ (D)×1.5 m(H)<br>Disengagement 10″ (D) ×10″(H)	S-301<br>Cyclone Hooper<br>Stain less steel 304<br>Body Dia 0.06 m	S-302/S-304<br>Inline Filters<br>Aluminum casing: SS wire mesh<br>Max Operating Pressure 40 bar<br>Max particle size 40 micron	S-303/S-305<br>Inline Filters<br>Aluminum casing: SS wire mesh<br>Max Operating Pressure 40 bar<br>Max particle size 3micron	E-401<br>Gas cooler<br>Shell &Tube Heat Exchanger<br>Carbon Steel<br>Exchange area<br>Approx. 0.8 m²	P-101<br>Gas Booster<br>Type: Air Driven Piston Pump<br>Max Outlet Pressure 55 bar	W-107<br>Cartridge Heater	

Figure S1. Globally unique pilot scale fluidised bed catalytic reactor schematic for propylene polymerization, designed and fabricated at Department of Chemical Engineering, University of Malaya, Malaysia.

© 2016 by the authors; licensee MDPI, Basel, Switzerland. This article is an open access article distributed under the terms and conditions of the Creative Commons by Attribution (CC-BY) license (http://creativecommons.org/licenses/by/4.0/).
